# Protease-activated receptor (PAR)-2 is required for PAR-1 signalling in pulmonary fibrosis

**DOI:** 10.1111/jcmm.12520

**Published:** 2015-02-16

**Authors:** Cong Lin, Jan von der Thüsen, Joost Daalhuisen, Marieke ten Brink, Bruno Crestani, Tom van der Poll, Keren Borensztajn, C Arnold Spek

**Affiliations:** a Center for Experimental and Molecular Medicine, Academic Medical CenterAmsterdam, The Netherlands; b Department of Pathology, Medisch Centrum HaaglandenDen Haag, The Netherlands; c Faculté de Médecine Paris 7 Diderot, Institut National de la Santé et de la Recherche MédicaleParis, France

**Keywords:** pulmonary fibrosis, protease-activated receptors (PARs), bleomycin

## Abstract

Idiopathic pulmonary fibrosis is the most devastating diffuse fibrosing lung disease of unknown aetiology. Compelling evidence suggests that both protease-activated receptor (PAR)-1 and PAR-2 participate in the development of pulmonary fibrosis. Previous studies have shown that bleomycin-induced lung fibrosis is diminished in both PAR-1 and PAR-2 deficient mice. We thus have been suggested that combined inactivation of PAR-1 and PAR-2 would be more effective in blocking pulmonary fibrosis. Human and murine fibroblasts were stimulated with PAR-1 and PAR-2 agonists in the absence or presence of specific PAR-1 or PAR-2 antagonists after which fibrotic markers like collagen and smooth muscle actin were analysed by Western blot. Pulmonary fibrosis was induced by intranasal instillation of bleomycin into wild-type and PAR-2 deficient mice with or without a specific PAR-1 antagonist (P1pal-12). Fibrosis was assessed by hydroxyproline quantification and (immuno)histochemical analysis. We show that specific PAR-1 and/or PAR-2 activating proteases induce fibroblast migration, differentiation and extracellular matrix production. Interestingly, however, combined activation of PAR-1 and PAR-2 did not show any additive effects on these pro-fibrotic responses. Strikingly, PAR-2 deficiency as well as pharmacological PAR-1 inhibition reduced bleomycin-induced pulmonary fibrosis to a similar extent. PAR-1 inhibition in PAR-2 deficient mice did not further diminish bleomycin-induced pulmonary fibrosis. Finally, we show that the PAR-1-dependent pro-fibrotic responses are inhibited by the PAR-2 specific antagonist. Targeting PAR-1 and PAR-2 simultaneously is not superior to targeting either receptor alone in bleomycin-induced pulmonary fibrosis. We postulate that the pro-fibrotic effects of PAR-1 require the presence of PAR-2.

## Introduction

Idiopathic pulmonary fibrosis (IPF) is a characteristic form of fibrosing idiopathic interstitial pneumonia which has a devastating prognosis 1,2. The therapeutic options are limited and, to date, only pirfenidone has been granted orphan drug status in the EU for the treatment of mild-to-moderate IPF 3. Although the understanding of IPF continues to evolve, the molecular mechanisms underlying the pathogenesis of IPF are still far from completely understood. The current paradigm postulates that the abnormal wound healing response to lung epithelial injury subsequently leads to pulmonary fibrosis 4. IPF lesions are distinctively characterized by the formation and proliferation of fibroblast foci in the background of excessive extracellular matrix (ECM) deposition 1,2,4. Therefore, unraveling the mechanisms by which fibroblasts replicate and secrete ECM proteins could be beneficial for conceiving effective therapeutic strategies 5.

Protease-activated receptors (PARs) belong to the superfamily of G-protein-coupled receptors (GPCRs) 6. Unlike other GPCRs, which are activated by ligand binding, PARs are irreversibly activated by proteolytic cleavage 7. After proteolytic activation of PARs, a novel tethered ligand is exposed that folds back over the receptor to trigger several downstream signalling pathways, contributing to a broad range of pathophysiological functions 6–10. Although blood factors are the archetypal activating proteases of PARs, it is now well-established that multiple proteases, such as thrombin, matrix metalloproteinase-1, factor (F)VII, FXa, trypsin and tryptase, can activate individual PARs with different affinity and trigger specific responses *via* biased agonist signalling 6–11.

In the context of lung injury and pulmonary fibrosis, accumulating evidence suggests that both PAR-1 and PAR-2 induce pro-inflammatory and pro-fibrotic processes that aggravate disease progression. PAR-1 activation enhances inflammation in the pulmonary epithelium, it induces the differentiation of fibroblasts into myofibroblasts and stimulates ECM synthesis 12–14. Moreover, genetic ablation of PAR-1 15, as well as pharmacological PAR-1 inhibition 16, limit bleomycin-induced acute lung inflammation and fibrosis, as evident from reduced total collagen level in the lung in combination with decreased levels of proinflammatory and profibrotic mediators, such as transforming growth factor (TGF)-β, interleukin (IL)-6 and monocyte chemoattractant protein-1. Furthermore, PAR-1 expression is increased within fibroproliferative and inflammatory foci in IPF patients 14. PAR-2 activation induces acute lung inflammation and also triggers fibroproliferative responses in fibroblasts, such as proliferation, migration and differentiation into myofibroblasts 17–19. In line, the absence of PAR-2 affords protection from bleomycin-induced pulmonary fibrosis, as evident from a reduction in the extent and severity of fibrotic lesions and diminished collagen expression 20. PAR-2 expression is also increased in lungs of IPF patients and its expression highly correlates with the extent of honeycombing 20–22.

Overall, these studies highlight PAR-1 and PAR-2 as critical contributors in promoting pulmonary fibrosis. Importantly, in the experimental bleomycin model, pulmonary fibrosis is not completely abolished in mice that harbour deficiency for either PAR-1 or PAR-2. Therefore, in this study, we have been suggested that the simultaneous inhibition of PAR-1 and PAR-2 would be superior to targeting either receptor alone in pulmonary fibrosis.

## Materials and methods

### Cells and reagents

Mouse embryonic NIH3T3 fibroblasts (American Type Culture Collection, Manassas, VA, USA; CRL-1658) and human lung fibroblast (HLFs from control lungs, isolated as described before 23) were cultured in DMEM supplemented with 10% foetal calf serum (FCS). Cells were grown at 37°C in an atmosphere of 5% CO_2_. Unless indicated otherwise, cells were washed twice with PBS and serum-starved for 4 hrs before stimulation. Thrombin (T7009; ≥1000 NIH Units/mg) and trypsin (T0303; 13,000–20,000 BAEE Units/mg) were from Sigma-Aldrich (St-Louis, MO, USA), whereas P1pal-12 (palmitate-RCLSSSAVANRS-NH2) 24 and P2pal-18s (palmitate-RSSAMDENSEKKRKSAIK-NH2) 25 were from GL Biochem Ltd (Shanghai, China). Both pepducins, which are insoluble in water, were dissolved in DMSO followed by dilutions in PBS or saline leading to final DMSO concentrations of 6% for the *in vivo* experiment and 0.1% for *in vitro* experiments.

### Western blot

Western blots were performed essentially as described before 19. In brief, cells were lysed in Laemmli lysis buffer and the lysates were incubated for 5 min. at 95°C. Afterwards, protein samples were separated by 10% SDS gel electrophoresis and transferred to a PVDF membrane (Millipore, Billerica, MA, USA). Membranes were blocked for 1 hr in 4% milk in TBST and incubated overnight with monoclonal antibodies against α-smooth muscle actin (a-SMA), tubulin, collagen (all Santa Cruz Biotechnology, Santa Cruz, CA, USA), phospho-ERK1/2 or total ERK1/2 (both Cell Signalling, Leiden, The Netherlands) at 4°C. All secondary antibodies were horseradish peroxidase (HRP)-conjugated from DakoCytomation (Glostrup, Denmark) and diluted according to the manufacturer's instructions. Blots were imaged using Lumilight plus ECL substrate from Roche (Almere, The Netherlands) on an ImageQuant LAS 4000 biomolecular imager from GE Healthcare (Buckinghamshire, UK).

### Wound scratch assay

Scratch assays were performed essentially as described before 19,26. Cells were seeded in six-well plates in DMEM supplemented with 10% FCS. After the cells formed a confluent monolayer, a scratch was created in the center of the monolayer by a sterile p200 pipette tip. Next, medium was removed and cells were washed with serum-free medium to remove floating debris. The cells were subsequently incubated for 18 hrs with serum-free medium (negative control), serum-free medium supplemented with 10 nM thrombin/trypsin or serum-free medium containing 10 nM thrombin/trypsin and 10 μM PAR-1 or PAR-2 antagonist (P1pal-12/P2pal-18s). When indicated, cells were pre-incubated with 10 μM pepducin for 30 min. before scratching. The ability of cells to close the wound was assessed by comparing the 0- and 18-hr phase-contrast micrographs of 6 marked points along the wounded area. The percentage of non-recovered wound area was calculated by dividing the non-recovered area after 18 hrs by the initial area at 0 hr as previously described.

### Animal model of pulmonary fibrosis

Wild-type C57Bl/6 mice were purchased from Charles River (Someren, The Netherlands). PAR-2 deficient (PAR-2-/-) C57Bl/6 mice were originally provided by Jackson Laboratories Bar Harbor (ME, USA) and bred at the animal care facility of the Academic Medical Center. All procedures were performed on 10-week-old mice in accordance with the Institutional Standards for Humane Care and Use of Laboratory Animals. Experiments were approved by the Animal Care and Use Committee of the Academic Medical Center (Amsterdam, The Netherlands).

Bleomycin (Sigma-Aldrich) was administered by intranasal instillation (1 mg/kg bw) under anaesthesia. We specifically opted for intranasal instillation instead of intratracheal instillation as the former administration route, which is also a well-recognized manner to induce pulmonary fibrosis, causes less discomfort to the mice and is therefore the preferred model of the Animal Welfare Committee of our institute. Bleomycin was instilled in 16 wild-type and 16 PAR-2 deficient mice. Per genotype, eight mice were subsequently treated with P1pal-12 (dissolved in 6% DMSO) whereas the other eight mice were treated with DMSO alone. The latter mice are indicated as solvent controls throughout the manuscript. Eight wild-type mice were instilled with saline instead of bleomycin were used as non-fibrotic controls and are indicated as saline controls. P1pal-12 (PAR-1 antagonist) was administered 30 min. before bleomycin administration and subsequently once daily until the end of the experiment at a dose of 2.5 mg/kg (based on previous dose finding experiments 16). Since the most suitable time-point for assessing lung fibrosis is day 14 after bleomycin challenge 27, mice were killed at this time-point, after which the left lung was taken for histology and the right one was homogenized.

### TGF- β ELISA

Transforming growth factor-β1 was measured with the Mouse TGF-beta 1 DuoSet kit (R&D Systems, UK Abingdon) as suggested by the manufacturer.

### Hydroxyproline assay

Hydroxyproline analysis was performed by the hydroxyproline assay kit as per the manufacturer's instructions (Sigma-Aldrich Zwijndrecht, The Netherlands) and as described before 16.

### (Immuno)Histological analysis

The excised lung was fixed in formalin, embedded in paraffin and 4-μm-thick slides were subsequently deparaffinized, rehydrated and washed in deionized water. Slides were stained with haematoxylin and eosin and Masson's trichrome according to routine procedures. As for the immunohistochemistry, 4-μm sections were first deparaffinized and rehydrated. Endogenous peroxidase activity was quenched with 0.3% H_2_O_2_ in methanol. Smooth muscle actin (α-SMA) and collagen staining were performed with an anti-α-SMA antibody (1:1000, 24 hr at 4°C, Santa Cruz Biotechnology) or an anti-collagen-I antibody (1:800, overnight at 4°C; GeneTex Irvine, CA, USA). A horseradish peroxidase-conjugated polymer detection system (Immunologic, Duiven, The Netherlands) was applied for visualization, using an appropriate secondary antibody and diaminobenzidine (DAB) staining. Slides were photographed with a microscope equipped with a digital camera (Leica Wetzlar, Germany CTR500).

Histological examination and Ashcroft score were performed as described before 18. Smooth muscle actin (α-SMA) staining was graded in a blinded fashion on a scale from 0 to 3 as described before 20. Pictures of collagen staining were taken to cover the entirety of all sections. Colour intensity of stained areas was analysed semi-quantitatively with ImageJ and expressed as percentage of the surface area essentially as described before 28.

### Statistics

Statistical analyses were conducted using GraphPad Prism (GraphPad software, San Diego, CA, USA). Comparisons between conditions were analysed using two tailed unpaired *t*-tests when the data were normally distributed; otherwise Mann–Whitney analysis was performed. Results are expressed as mean ± SEM, *P* < 0.05 are considered significant.

## Results

### PAR-1 and PAR-2 activating proteases induce pro-fibrotic responses

**Figure 1:**
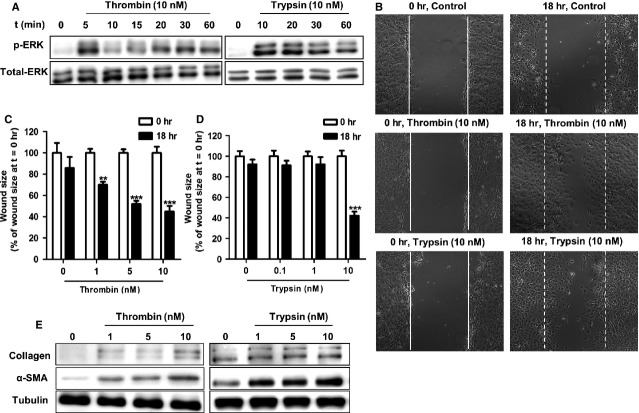
Thrombin and trypsin induce signalling and pro-fibrotic responses in NIHT3T3 fibroblasts. (A) Western blot analysis of ERK1/2 phosphorylation in NIH3T3 cells after stimulation with 10 nM thrombin (left) or with 10 nM trypsin (right). Total ERK served as loading control. (B) Wound closure of NIH3T3 fibroblast monolayers after treatment with PBS (control, top panel), thrombin (10 nM; middle panel) or trypsin (10 nM; bottom panel) for 18 hrs. Shown are photographs of representative microscopic fields. Quantification of wound closure induced by thrombin (C) or trypsin (D) as described in Materials and methods. Data are expressed as mean ± SEM (*n* = 6). **P < 0.01, ***P < 0.001. (E) Western blot analysis of α-SMA and collagen expression in NIH3T3 cells 24 hrs after stimulation with the indicated concentrations of thrombin or trypsin. Tubulin served as a loading control.

Protease-activated receptor-1 is prototypically activated by thrombin whereas trypsin is the best characterized PAR-2 agonist. Compelling evidence shows that PAR stimulation of fibroblasts leads to the phosphorylation of extracellular signal-regulated kinase (ERK)1/2(a surrogate marker for PAR-1 and PAR-2 activation), cell migration, differentiation into myofibroblasts and ECM synthesis 12–19. We previously showed that NIH3T3 cells express functional PAR-1 and PAR-2 19 and here we first validated the efficacy of thrombin and trypsin to induce these cellular responses. As shown in Figure1A, both thrombin (10 nM) and trypsin (10 nM) induced ERK1/2 activation in murine NIH3T3 fibroblasts. In wound scratch assays, thrombin treatment led to wound closure in a dose-dependent manner (Fig.1B and C), whereas only the highest concentration of trypsin strongly induced wound closure by about 60% compared to solvent treated cells (Fig.1B and D). Furthermore, both thrombin and trypsin-induced fibroblast differentiation (reflected by increased α-SMA expression) and collagen synthesis (Fig.1E). These data thus indicate that thrombin and trypsin both can induce pro-fibrotic responses in NIH3T3 fibroblasts. On the basis of these data, we opted to use 10 nM of thrombin and trypsin in our subsequent experiments.

### Simultaneous stimulation of both PAR-1 and PAR-2 does not show additive pro-fibrotic effects

**Figure 2:**
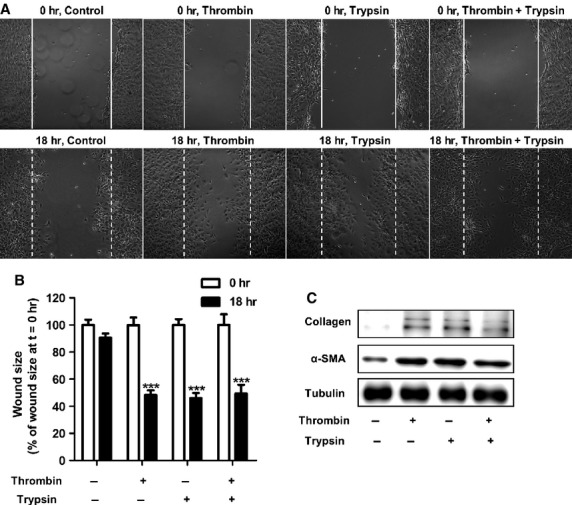
Simultaneous activation of PAR-1 and PAR-2 on NIH3T3 fibroblasts does not trigger additive pro-fibrotic effects. (A) Wound size of NIH3T3 fibroblast monolayers after treatment with PBS (control), thrombin (10 nM), trypsin (10 nM) or the combination of 10 nM thrombin and 10 nM trypsin for 18 hrs. Shown are photographs of representative microscopic fields. (B) Quantification of the results depicted in (A) as described in the Materials and methods section. Data are expressed as mean ± SEM (*n* = 6), ***P < 0.001. (C) Western blot analysis of α-SMA and collagen in NIH3T3 cells 24 hrs after stimulation with PBS (control), thrombin (10 nM), trypsin (10 nM) or combination thereof. Tubulin served as a loading control.

After having established that both PAR-1 and PAR-2 promote pro-fibrotic responses in fibroblasts, we next assessed whether simultaneous activation of PAR-1 and PAR-2 induces a more robust pro-fibrotic response by stimulating cells with thrombin and trypsin at the same time. Interestingly, as shown in Figure2A and B, no additive effect could be observed on wound closure. Wound sizes were decreased by approximately 50% in cells treated with thrombin, trypsin or a combination of thrombin and trypsin. Likewise, combined thrombin and trypsin treatment did not induce higher α-SMA and collagen expression than that observed after single PAR agonist treatment (Fig.2C). Interestingly, delayed trypsin treatment (either 2, 4, 8 or 12 hrs after thrombin stimulation) still did not show any additive effect on thrombin-induced wound healing and/or fibrotic marker expression (Fig. S1A and C).

### PAR-1 inhibition in PAR-2 deficient mice does not further limit pulmonary fibrosis *in vivo*

**Figure 3:**
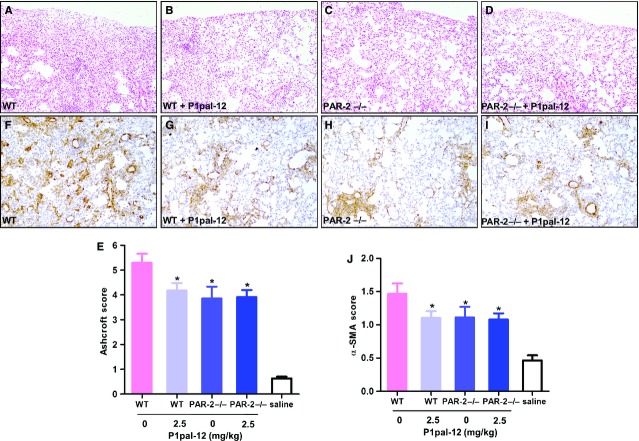
Pharmacological PAR-1 inhibition in PAR-2 deficient mice does not further reduce pulmonary fibrosis (A–D, ×100). Representative haematoxylin and eosin staining on lung tissue sections obtained 14 days after bleomycin instillation in wild-type mice (A), wild-type mice treated with 2.5 mg/kg P1pal-12 (B), PAR-2 deficient mice (C) or PAR-2 deficient mice treated with 2.5 mg/kg P1pal-12 (D). (E) Quantification of pulmonary fibrosis using the Ashcroft score (F–I, ×100). Representative pictures of α-SMA deposition in the lungs of wild-type mice (F), wild-type mice treated with 2.5 mg/kg P1pal-12 (G), PAR-2 deficient mice (H) or PAR-2 deficient mice treated with 2.5 mg/kg P1pal-12 (I). (J) Quantification of pulmonary α-SMA deposition as described in the Materials and methods section. Mice not instilled with bleomycin are indicated as saline. Data are expressed as mean ± SEM (*n* = 8 per group). *P < 0.05. Note that all mice were bleomycin-treated.

In previous experiments, we showed that blocking PAR-1 by P1pal-12 limits bleomycin-induced pulmonary fibrosis in a dose-dependent manner 16. Here, we applied the optimal P1pal-12 dose (2.5 mg/kg once daily) to treat both wild-type and PAR-2 deficient mice, and compared bleomycin-induced fibrosis with solvent control treated wild-type and PAR-2 deficient mice. As shown in Figure3, extensive patchy areas of fibrosis were formed 14 days after bleomycin instillation in solvent treated wild-type mice, accompanied by a marked accumulation of inflammatory cells and significant ECM deposition (Fig.3A). Both P1pal-12 treatment and PAR-2 deficiency significantly reduced the severity of regional interstitial fibrosis as assessed by the Ashcroft score (reduction in approximately 22% and 27% respectively, Fig.3B, C, E). Surprisingly, PAR-2 deficient mice treated with the PAR-1 antagonist P1pal-12 did not show a further reduction in fibrosis as that observed in solvent treated PAR-2 deficient mice (about 26% reduction; Fig.3D and E).

To substantiate our findings that PAR-1 inhibition does not further decrease fibrosis in PAR-2 deficient mice, we next analysed α-SMA expression immunohistochemically. A considerable increase in α-SMA expression was seen in focal fibrotic lesions of solvent treated wild-type mice upon bleomycin instillation (Fig.3F). Both pharmacological PAR-1 inhibition and genetic PAR-2 ablation significantly attenuated bleomycin-induced α-SMA expression (Fig.3G, H, J). Again, PAR-1 inhibition in PAR-2 deficient mice was not superior to either PAR-1 inhibition or PAR-2 deficiency alone (Fig.3I and J).

**Figure 4:**
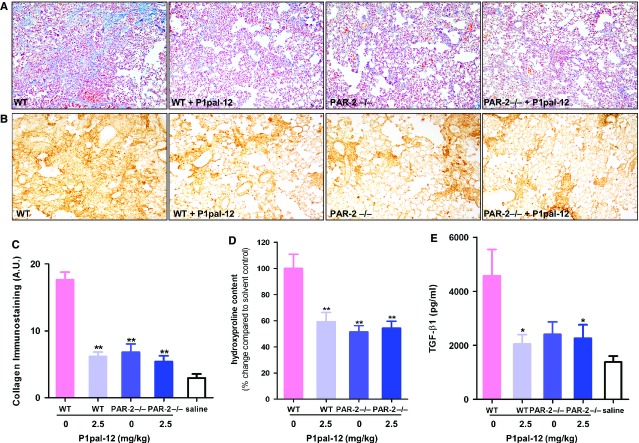
PAR-1 inhibition in PAR-2 deficient mice does not attenuate collagen deposition and active-TGF-β production to a greater extent than single receptor targeting. Representative pictures (×100) of (A) Masson-trichrome and (B) collagen stained lung sections obtained 14 days after bleomycin instillation. (C) Quantification of collagen immunostaining in the different groups of mice (semi-quantitative image analysis). Hydroxyproline content (D) and TGF-β1 levels (E) in lung homogenates of the different groups of mice obtained 14 days after saline or bleomycin instillation. Mice not instilled with bleomycin are indicated as saline. Data are expressed as mean ± SEM (*n* = 8 per group). *P < 0.05, **P < 0.01.

We next analysed collagen deposition in the lungs. As shown in Figure4A–C, Masson-trichrome and collagen I analysis showed similar reductions of collagen deposition in P1pal-12 (PAR-1 antagonist) treated wild-type mice, solvent control treated PAR-2 deficient mice or P1pal-12 treated PAR-2 deficient mice. In line, compared with bleomycin-instilled solvent treated wild-type mice, hydroxyproline levels decreased by 41 ± 7%, 49 ± 5% and 46 ± 5% in P1pal-12 treated wild-type mice, solvent control treated PAR-2 deficient mice and P1pal-12 treated PAR-2 deficient mice respectively (Fig.4D).

Transforming growth factor-β1 is one of the most important pro-fibrotic mediators and its expression is frequently associated with PAR regulation in fibrotic diseases 29. We therefore assessed TGF-β1 levels in lung homogenates of saline or bleomycin-instilled mice. As shown in Figure4E, TGF-β1 levels increased around twofold in solvent treated bleomycin-instilled wild-type mice compared with saline treated controls. Again, the increase in TGF-β1 was attenuated in PAR-2 deficient and P1pal-12 treated wild-type or PAR-2 deficient mice. Altogether, these data show that the combined inhibition of PAR-1 and PAR-2 also has no additive effect *in vivo*.

### PAR-2 is required for PAR-1-induced pro-fibrotic responses in fibroblasts

**Figure 5:**
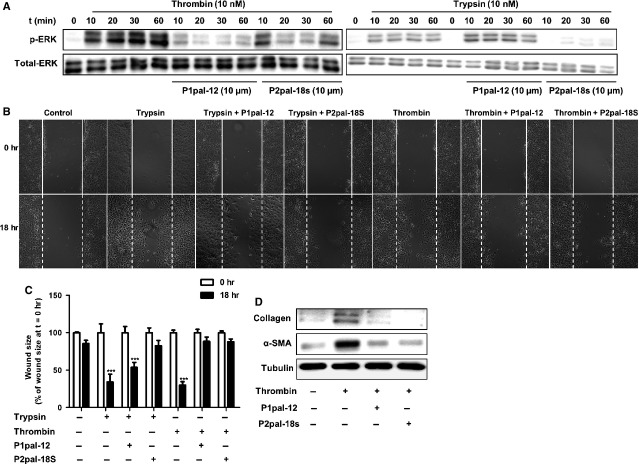
PAR-2 is required for PAR-1-induced pro-fibrotic responses. (A) Western blot analysis of ERK1/2 phosphorylation in NIH3T3 cells after stimulation with trypsin (10 nM) or thrombin (10 nM), in the absence (-) or presence (+) of P1pal-12 (10 μM) or P2pal-18s (10 μM). P1pal-12 or P2pal-18 sec. was added 30 min. before the stimulation. Total ERK served as loading control. (B) Wound size of NIH3T3 fibroblast monolayers after treatment with DMSO (control), trypsin (10 nM) or thrombin (10 nM) for 18 hrs in the presence or absence of P1pal-12 or P2pal-18s. Cells were pre-incubated with 10 μM P1pal-12 or P2pal-18s for 30 min. as indicated. Shown are photographs of representative microscopic fields. (C) Quantification of the results depicted in (B) as described in the Materials and methods section. Data are expressed as mean ± SEM (*n* = 6). ***P < 0.001. (D) Western blot analysis of α-SMA and collagen expression in NIH3T3 cells 24 hrs after stimulation with DMSO (control) or thrombin (10 nM), in the presence (+) or absence (-) of P1pal-12 (10 μM) or P2pal-18s (10 μM). Tubulin served as a loading control.

Our data so far show that combined activation of PAR-1 and PAR-2 is just as effective as single PAR activation on promoting fibrotic responses in fibroblasts. Moreover, simultaneous inhibition of PAR-1 and PAR-2 was not superior to targeting either receptor alone *in vivo,* suggesting that PAR-1 and PAR-2 may actually act in concert to promote fibrosis. Consequently, we analysed PAR agonist-induced pro-fibrotic responses in fibroblasts in the absence or presence of specific PAR-1 (P1pal-12) or PAR-2 (P2pal-18s) inhibitors. As shown in Figure5A, thrombin-induced ERK1/2 phosphorylation was largely inhibited in the presence of P1pal-12. Surprisingly, however, thrombin-induced ERK1/2 phosphorylation is also inhibited by the PAR-2 inhibitor P2pal-18s. In contrast, trypsin-induced ERK1/2 activation is only inhibited by P2pal-18s but not by P1pal-12 treatment (Fig.5A). In wound scratch assays, P1pal-12 pre-treatment blocked thrombin induced wound closure but only slightly reduced trypsin-induced closure, whereas P2pal-18s pre-treatment completely inhibited both trypsin and thrombin induced wound closure (Fig.5B and C). Consistent with these results, thrombin induced α-SMA and collagen expression was significantly down-regulated in P2pal-18s-pre-treated cells (Fig.5D). In addition, delayed P2-pal-18s treatment was less efficient as compared to pre-treatment, as evident from a gradual decrease in preventing wound healing and fibrotic marker expression over time (Fig. S1B and D). These data suggest that once the signalling pathways are activated additional PAR-2 activation is irrelevant. Overall, PAR-1-induced responses in fibroblasts are blocked by a PAR-2 specific antagonist, suggesting that the presence of PAR-2 is required for PAR-1 dependent pro-fibrotic signalling.

### PAR-2 is also pivotal for PAR-1 to induce pro-fibrotic effects in HLFs

**Figure 6:**
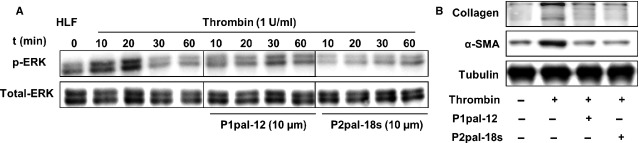
PAR-2 is required for PAR-1-mediated pro-fibrotic responses in HLFs. (A) Western blot analysis of ERK1/2 phosphorylation for the indicated time-points in HLFs after stimulation with thrombin (10 nM) in the absence (−) or presence (+) of P1pal-12 (10 μM) or P2pal-18s (10 μM). P1pal-12 or P2pal-18s was added 30 min. before the stimulation. Total ERK served as loading control. (B) Western blot analysis of α-SMA and collagen expression in HLFs 24 hrs after stimulation with either DMSO (control) or thrombin (10 nM) in the absence (−) or presence (+) of P1pal-12 (10 μM) or P2pal-18s (10 μM). Tubulin served as a loading control.

Finally, we aimed to confirm our *in vitro* findings using primary HLFs derived from (non-fibrotic) patients. As shown in Figure6A, stimulation of HLFs with thrombin induced ERK1/2 phosphorylation, which was blocked by pre-treatment with the PAR-1 antagonist P1pal-12 but also by pre-treatment with the PAR-2 antagonist P2pal-18s. Furthermore, thrombin-induced differentiation of HLFs into myofibroblasts (as assessed by a-SMA expression) and collagen production were also inhibited by P2pal-18s (Fig.6B), indicating that pro-fibrotic effects of PAR-1 in HLFs also require the presence of PAR-2.

## Discussion

Compelling evidence suggests that aberrant wound healing caused by acute lung injury may play a pathophysiological role in IPF. It has been documented that many proteases exert pro-inflammatory and pro-fibrotic effects by proteolytically activating PAR-1 and/or PAR-2 12,14,19–21. Even more importantly, preclinical experimental data show that mice lacking either receptor are protected against bleomycin-induced pulmonary fibrosis 15,20. However, bleomycin-induced pulmonary fibrosis was not completely diminished by pharmacological inhibition of PAR-1 16 or genetic ablation of either PAR-1 or PAR-2 15,20. In the current study, we aimed to assess whether PAR-1 and PAR-2 synergically promote fibrosis progression and thus whether the simultaneous inhibition of PAR-1 and PAR-2 would more efficiently limit pulmonary fibrosis as compared to single receptor inhibition. Strikingly, we show that, both *in vitro* and *in vivo,* the simultaneous stimulation or inhibition of PAR-1 and PAR-2 does not lead to additive effects. In fact, we show that the pro-fibrotic effects induced by PAR-1 stimulation require the presence of PAR-2.

The most interesting finding of our current study is the fact that PAR-2 is pivotal for PAR-1-induced fibrotic processes. We show that pharmacological inhibition of PAR-1 does inhibit bleomycin-induced fibrosis in wild-type mice but does not further diminish bleomycin-induced fibrosis in PAR-2 deficient mice, as evident from similar reductions in Ashcroft score, α-SMA expression and hydroxyproline content in the lungs. We unravelled the molecular basis for these findings *in vitro*. We show that PAR-1 dependent fibroblast migration, differentiation and ECM production is abolished in the presence of the specific PAR-2 inhibitor P2pal-18s (Fig.5) and we further confirmed these findings in HLFs. Overall, these results indicate that PAR-2 modulates the activity of PAR-1 thereby inducing pro-fibrotic responses.

**Figure 7:**
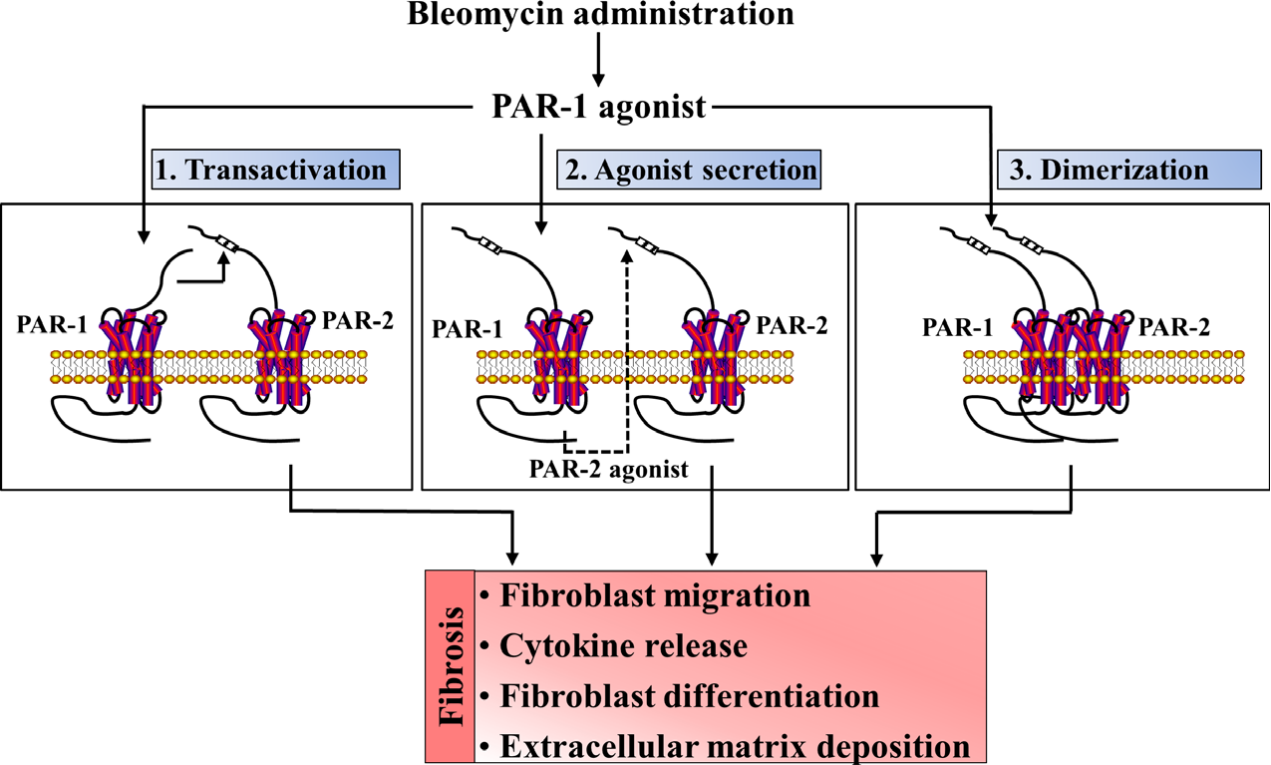
Schematic overview of potential mechanisms by which PAR-1 and PAR-2 act in concert to contribute to pulmonary fibrosis. Bleomycin administration leads to the release of a PAR-1 agonist that subsequently activates PAR-1 on fibroblasts. This activation may subsequently lead to transactivation of PAR-2 or to the production of a PAR-2 agonist thereby inducing pro-fibrotic processes like migration, differentiation and extracellular matrix deposition. As elaborated in the discussion section, however, most likely the PAR-1 agonist activates PAR-1/PAR-2 heterodimers thereby inducing the pro-fibrotic responses.

In recent years, several studies showed that PAR-1 and PAR-2 might facilitate each other's activity in different pathophysiological processes 30. For instance, protective effects of PAR-1 during sepsis require transactivation of PAR-2 signalling pathways 31, while PAR-2 regulates the PAR-1 hyperplastic response to arterial injury leading to stenosis 32. Moreover, in tumour biology it is shown that thrombin-induced melanoma cell migration and metastasis are dependent on both PAR-1 and PAR-2 activation 33. Finally, mammary adenocarcinoma cells lacking PAR-2 failed to express PAI-1 in response to thrombin activation 34, and a very recent study shows that PAR-1 and PAR-2 act as a functional unit in breast cancer development 35. Here, we extend these observations by showing cooperative signalling between PAR-1 and PAR-2 in the setting of pulmonary fibrosis (Fig.7).

The mechanism by which PAR-1 interacts with PAR-2 signalling in fibrosis remains elusive. Interestingly, several potential mechanisms have been suggested (excellently reviewed in Ref. 30). First, it has been described that the thrombin-generated tethered ligand of PAR-1 may transactivate PAR-2 36,37. However, P1pal-12 (PAR-1 antagonist) does not prevent thrombin-induced PAR-1 cleavage. Indeed, it is a cell-penetrating pepducin derived from the third intracellular loop of PAR-1 that – once inserted into the plasma membrane- interferes with interaction between the receptor and its G-proteins thereby blocking PAR-1 dependent signalling 24. Consequently, PAR-2 transactivation by the PAR-1 tethered ligand seems not to be the main mechanism in the present setting. An alternative explanation could be that PAR-1 activation induces the expression of a PAR-2 ligand that would subsequently induce fibrosis in a PAR-2 dependent manner. However, this explanation is not very likely because PAR-1 dependent ERK1/2 phosphorylation, which is also partially blocked by PAR-2 inhibition, occurs within minutes. It is difficult to envision that PAR-2 ligands are synthesized during this short time frame. Moreover, conditioned medium of thrombin-treated fibroblasts did not induce PAR-2 dependent fibrotic effects (data not shown). Finally, PAR-1 and PAR-2 may directly interact and form heterodimers that induce different signalling pathways compared to those induced by monomers 31,38. In line with such a mechanism, PAR-2 expression is low in quiescent lung fibroblasts but may considerably increase under inflammatory and fibrotic conditions thereby favoring the formation of PAR-1/PAR-2 complexes. Indeed, while PAR-1 expression remains constant on normal and IPF-derived fibroblasts, PAR-2 expression is low in normal fibroblasts but undergoes a dramatic up-regulation in IPF-derived fibroblasts 39. In line, bleomycin instillation induced PAR-2, and also PAR-1, mRNA expression levels increase in our experimental animals (Fig. S2). In addition, TGF-β stimulations increase PAR-2 levels both on the mRNA and protein level 21,40 and treatment with thrombin results in an up-regulation of PAR-2 mRNA level (data not shown). It is tempting to speculate that this latter notion also explains our observation that PAR-2 inhibition by P2pal-18 only partially blocked thrombin-induced ERK1/2 phosphorylation. The rapid phosphorylation of ERK (within minutes) may still largely be induced by PAR-1 monomers as the PAR-1/PAR-2 complexes have not yet been formed in large quantities 30. Irrespective the actual mechanism, our data strongly suggest that PAR-1-induced fibrosis is dependent on PAR-2 signalling.

Several issues should be kept in mind when interpreting our data. First, we used a single dose bleomycin model to induce pulmonary fibrosis. Although this model is sometimes criticized not to completely mimic the progression of fibrosis in IPF patients 41, this model shows typical histological patterns, like patchy parenchymal inflammation and interstitial fibrosis, as observed in IPF patients. A recent paper actually shows that bleomycin induces clinically meaningful molecular responses in the lungs of mice mimicking those occurring in the lungs of IPF patients (even in a quantitative manner) 42. Interestingly, the single dose bleomycin model was shown to be as effective in terms of producing a more substantial or progressive fibrotic response in the lungs as compared to a model of repetitive bleomycin exposures 42, which has been argued to be superior of the single dose model 43. Although there was no significant advantage in using the repetitive bleomycin model instead of the single challenge model, future studies using alternative fibrosis models should obviously validate our findings. Second, as thrombin also activates PAR-4, one may suggest that PAR-4 could also be involved in thrombin-induced fibrosis both *in vitro* and *in vivo*. However, PAR-4 is not expressed by HLFs 39 and several studies show that PAR-4 does not show pro-fibrotic effects after its activation 44. In addition, we previously showed that PAR-4 does not modify bleomycin-induced pulmonary fibrosis 45. The observed effects can consequently not depend on PAR-4. Moreover, PAR-1 agonist peptide showed similar responses as thrombin (Fig. S3) and the thrombin-induced responses are (almost) completely inhibited by a specific PAR-1 antagonist all suggesting thrombin induces fibrosis in a PAR-1 dependent and PAR-4 independent manner. Third, P1pal-12 (PAR-1 antagonist) treatment was started before bleomycin instillation and one could argue that delayed PAR-1 inhibition may alter our results. However, we previously showed that administration of P1pal-12 at different time-points after bleomycin instillation (*i.e*. either after 1 or 7 days) had similar effects in limiting the development of pulmonary fibrosis as compared to when administration was started before bleomycin instillation 16. Fourth, pharmacological inhibition of PAR-1 signalling and genetic ablation of PAR-2 either alone or in combination did significantly reduce pulmonary fibrosis but did not completely prevent fibrosis. Although reducing fibrosis or slowing down its progression may be clinically relevant, future studies need to establish whether PARs are prime candidates for the treatment of pulmonary fibrosis. Irrespective the potential clinical relevance, we highlight a cooperative contribution of PAR-1 and PAR-2 to pulmonary fibrosis.

In conclusion, the simultaneous inhibition of PAR-1 and PAR-2 is not superior to targeting either receptor alone in limiting pulmonary fibrosis. In fact, both *in vitro* and *in vivo*, we show that the pro-fibrotic effects induced by PAR-1 require the presence of PAR-2.

## Funding

This work was supported by grants from TiPharma (T1-215-1) and the Netherlands Organisation for Scientific Research (016.136.167).

## Conflicts of interest

The authors confirm that there are no conflicts of interest.

## Supporting Information

**Figure S1** (A and B) Wound size of NIH3T3 fibroblast monolayers after treatment with PBS (control), thrombin (10 nM) or thrombin in combination with trypsin (10 nM) (A) or P2pal-18s (10 μM) (B) for 18 hrs. **Figure S2** mRNA expression levels of PAR-1 and PAR-2 in lung homogenates of wild-type mice obtained 14 days after bleomycin or saline instillation. Data are expressed as mean ± SEM (*n* = 8). **Figure S3** (A) Quantification of wound closure of NIH3T3 fibroblast monolayers induced by PAR-1 agonist peptide or thrombin as described in Mater.
