# Supplementary material for: Protease-activated receptor (PAR)-2 is required for PAR-1 signalling in pulmonary fibrosis
Source: J Cell Mol Med. 2015 Feb 16;19(6):1346–56. doi: 10.1111/jcmm.12520 (PMC4459848; doi:10.1111/jcmm.12520)
Supplement: Supplementary file 1 [file jcmm0019-1346-sd1.doc]

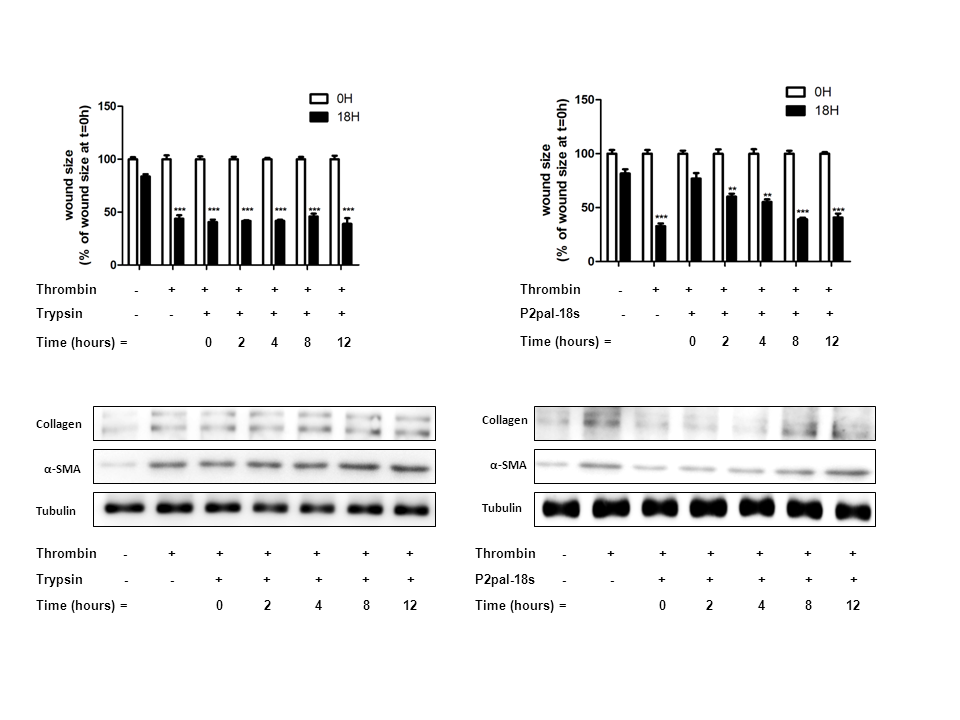


**A**

**B**

**C**

**D**

*Supplementary figure 1:* (**A-B**) Wound size of NIH3T3 fibroblast monolayers after treatment with PBS (control), thrombin (10 nM) or thrombin in combination with trypsin (10 nM; (**A**)) or P2pal-18s (10 µM; (**B**)) for 18 hours. Trypsin or P2pal-18s treatment was started simultaneously with thrombin treatment (indicated as t=0, or at different time points after thrombin stimulation (either 2, 4, 8 or 12 hours)). Data are expressed as mean±SEM (n=6). ** P<0.01, *** P<0.001. (**C-D**) Western blot analysis of α-SMA and collagen expression in NIH3T3 cells 24 hours after stimulation with thrombin or thrombin in combination with trypsin (**C**) or P2pal-18s (**D**). Tubulin served as a loading control. The timing of trypsin or P2pal-18s treatment is indicated in the figure.

*Supplementary figure 2:* mRNA expression levels of PAR-1 and PAR-2 in lung homogenates of wildtype mice obtained 14 days after bleomycin or saline instillation. Data are expressed as mean±SEM (n=8).


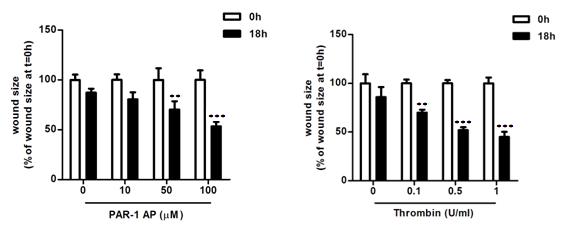


A

B

**
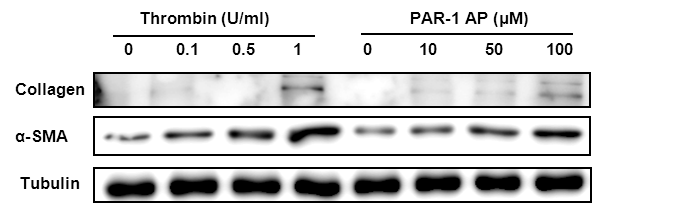
**

*Supplementary figure 3:* (**A**) Quantification of wound closure of NIH3T3 fibroblast monolayers induced by PAR-1 agonist peptide or thrombin as described in Materials and Methods. Data are expressed as mean±SEM (n=6). ** P<0.01, *** P<0.001. (**B**) Western blot analysis of collagen and α-SMA expression in NIH3T3 cells 24 hours after stimulation with the indicated concentrations of thrombin or PAR-1 agonist peptide. Tubulin served as a loading control.
